# Combining Multiple Omics with Molecular Dynamics Reveals SCP2-Mediated Cytotoxicity Effects of Aflatoxin B1 in SW480 Cells

**DOI:** 10.3390/toxins16090375

**Published:** 2024-08-24

**Authors:** Mengting Chen, Jiaxin Wen, Yiyan Qiu, Xinyue Gao, Jian Zhang, Yifan Lin, Zekai Wu, Xiaohuang Lin, An Zhu

**Affiliations:** 1 Key Laboratory of Gastrointestinal Cancer (Fujian Medical University), Ministry of Education, Fuzhou 350108, China; 2 Jimei District Center for Disease Control and Prevention, Xiamen 361022, China; 3 School of Public Health, Fujian Medical University, Fuzhou 350108, China

**Keywords:** aflatoxin B1, multiple omics, intestinal toxicity, oxidative stress, mitochondrial damage, lipid metabolism disorder

## Abstract

Aflatoxins belong to a class of mycotoxins, among which aflatoxin B1 (AFB1) has detrimental effects on the health of both animals and humans. It is associated with long-term exposure-induced carcinogenicity, hepatotoxicity, renal toxicity, neurotoxicity, and immunosuppressive properties, resulting in a variety of diseases. The intestine is the first barrier for human exposure to AFB1, but limited investigations have been conducted to clarify the underlying mechanisms of intestinal cytotoxicity. The mechanism of AFB1-induced cytotoxicity was investigated in this study using an integrated approach combining transcriptome, proteome, and metabolome analysis along with molecular dynamics simulation. After exposing SW480 cells to 50 μM AFB1 for 72 h, the transcriptome, proteome, and metabolome exhibited significant enrichment in pathways associated with oxidative stress, fatty acid and lipid metabolism, and glutathione metabolism. The experimental results demonstrated that AFB1 significantly reduces SW480 cells viability, and induces oxidative stress, calcium overload, mitochondrial damage, and lipid metabolism disorders.

## 1. Introduction

Aflatoxins are secondary metabolites produced by *Aspergillus flavus* and *Aspergillus parasitic*. Among them, aflatoxin B1 (AFB1) is one of the most widely distributed and toxic variants, and was classified as a Group I carcinogen by the World Health Organization (WHO) in 1993 [1]. The presence of AFB1 is commonly detected in various crops, including cereals, peanuts, maize, and their by-products, throughout the entire growth cycle as well as during transportation and storage processes [2]. The acceptable limits of AFB1 in food for human consumption and animal feed have been reported to be 40 parts per billion (ppb) and 300 ppb, respectively [3]. The consumption of feed contaminated with AFB1 significantly impairs animal performance and health [4,5]. Therefore, AFB1 contamination poses one of the most critical problems threatening food safety.

Transcriptomics is the technology that focuses on the types, abundance, and spatiotemporal expression patterns of mRNA, which is transcribed from DNA [6]. In 2001, the human genome sequence, covering more than 96% of the euchromatic regions, was completed roughly through the cooperation of 20 groups [7]. Since then, model organisms such as mice [8], rats [9], and chimpanzees [10] were sequenced to build genome sequences, which have laid the foundation for subsequent research. Recently, Wu et al. [11] proved that the reproductive toxicity of oridonin was induced by the tight junction, mitochondrial damage, and the Wnt signaling pathway, utilizing high-throughput sequencing and various genome databases. Therefore, transcriptomics has not only deepened the understanding of gene function and regulatory mechanisms but also provided insights into the molecular-level responses of organisms to environmental changes, developmental processes, and disease.

Proteomics has made remarkable progress in recent years, which has greatly expanded its applications in biomedicine [12]. The technology makes it possible to specifically identify and measure the expression levels of multiple proteins in biological samples, providing valuable information for the recognition of disease mechanisms and biomarkers. Furthermore, proteomics has been increasingly utilized in the study of fungal toxicity mechanisms. Previous research has revealed the antifungal mechanism of copper through proteomic analysis; namely, copper stress down-regulated protein synthesis and up-regulated protein degradation in *Cryptococcus neoformans*, thereby inhibiting its growth [13]. In another study, the potential mechanism underlying the damage to intestinal epithelial integrity caused by the combination of aflatoxin M1 (AFM1) and ochratoxin A was analyzed using integrated transcriptomics and proteomics [14].

Metabolomics has received considerable attention as a novel experimental approach for monitoring dynamic changes in metabolites and identifying key targets to elucidate the mechanisms underlying metabolic processes [15]. Metabolomics analysis has been widely applied in the study of diverse diseases, like cardiovascular diseases [16], neurodegenerative diseases [17], and digestive diseases [18]. The metabolomics results of male F344 rats gavaged with 75 µg/kg AFB1 for 5 weeks demonstrate that AFB1 disrupts the production of fatty acids, amino acids, and carbohydrates, leading to intestinal toxicity [19]. In addition, the metabolomics analysis of NCM460 cells treated with 2.5 µM AFB1 for 24 h revealed that AFB1 disrupted intestinal homeostasis by significantly increasing the content of glycerophospholipids, thereby exerting enteric toxicity [20].

The intestinal tract not only acts as the main location for nutrient digestion and absorption, but also functions as a vital barrier against toxins and detrimental substances. Consequently, it is common to observe higher concentrations of mycotoxins within intestinal cells compared to other tissues [21]. The presence of AFB1 induces intestinal injury across different species, as it alters the structure of intestinal epithelial cells and compromises the integrity of the intestinal barrier, ultimately resulting in a loss of barrier function [4]. Prolonged exposure to a diet with a low level of AFB1 resulted in a reduction in the digestibility of the total intestine and destroyed the integrity of the pig intestinal barrier by diminishing its antioxidant capacity and promoting proinflammatory cytokine production [22]. In this investigation, AFB1 was used to treat SW480 cells to study potential mechanisms of cytotoxicity via multi-omics and molecular dynamics.

## 2. Result

### 2.1. Cell Viability

**Figure 1:**
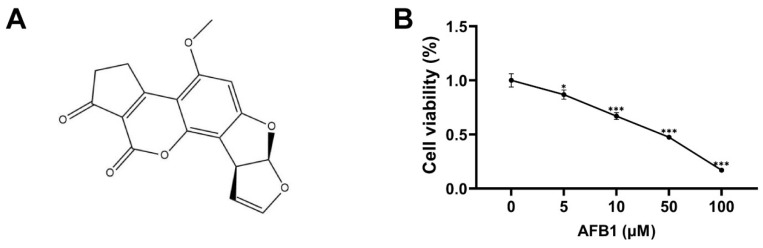
(**A**) The chemical structure of AFB1. (**B**) The viability of SW480 cells following treatment with 0, 5, 10, 50, and 100 μM AFB1 for 72 h. The data were presented as the mean ± SD. * *p* < 0.05, *** *p* < 0.001, n = 3.

The cell viability was determined via the MTT assay following the culture of SW480 cells with diverse doses of AFB1 for 72 h (Figure 1B). Compared to the control group, cell viability was reduced to 86.8%, 67.0%, 47.5%, and 16.9% with an increase of 5, 10, 50, and 100 μM AFB1.

### 2.2. Transcriptomic Analysis Based on Differentially Expressed Genes (DEGs)

**Figure 2:**
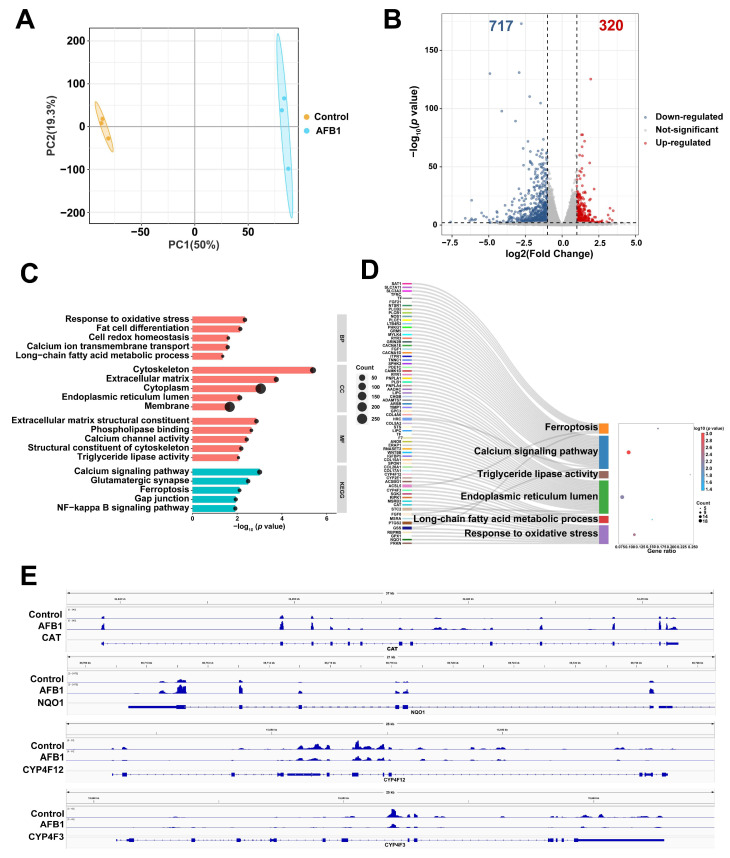
(**A**) Performing PCA on mRNA expression in the control and AFB1 groups. (**B**) Volcano plot shows a total of 1037 differential genes, 320 up-regulated and 717 down-regulated between the two groups, were screened. (**C**) The GO and KEGG enrichment of DEGs. (**D**) The Sankey diagram of ferroptosis, calcium signaling pathway, triglyceride lipase activity, endoplasmic reticulum lumen, long-chain fatty acid metabolic process, and response to oxidative stress pathways. (**E**) The level of *CAT*, *NQO1*, *CYP4F12*, and *CYP4F3* mRNA transcripts observed by IGV.

The transcriptomic analysis was performed to investigate the alterations in gene function associated with AFB1 treatment in SW480 cells. The control group can be distinguished from the AFB1 group based on principal component analysis (PCA) score plots (Figure 2A). A total of 13,850 genes were detected and matched based on gene symbols through mRNA-seq. The differential expression analysis comparing the control group with the AFB1 group revealed a total of 1037 DEGs meeting the criteria of a fold change >2 or <0.5 and a *p* value < 0.05. The expression levels of 320 genes were found to be up-regulated, while those of 717 genes were down-regulated among these DEGs (Figure 2B). We performed a gene ontology (GO) and Kyoto Encyclopedia of Genes and Genomes (KEGG) enrichment analysis of the DEGs. The DEGs in the control group and AFB1 group were mainly enriched in response to oxidative stress, long-chain fatty acid metabolic process, endoplasmic reticulum lumen, triglyceride lipase activity, calcium signaling pathway, and ferroptosis (Figure 2C). The Sankey diagram presented in Figure 2D illustrates the specific genes that exhibit enrichment under the GO and KEGG entries mentioned above. We analyzed the *NQO1* and *CAT* genes enriched in response to oxidative stress and the *CYP4F3* and *CYP4F12* genes enriched in the long-chain fatty acid metabolic process to explore their specific changes. The integrative genomics viewer (IGV) plots (Figure 2E) showed the up-regulation of *CAT* and *NQO1*, while a down-regulation of *CYP4F12* and *CYP4F3* was observed following AFB1 treatment.

**Figure 3:**
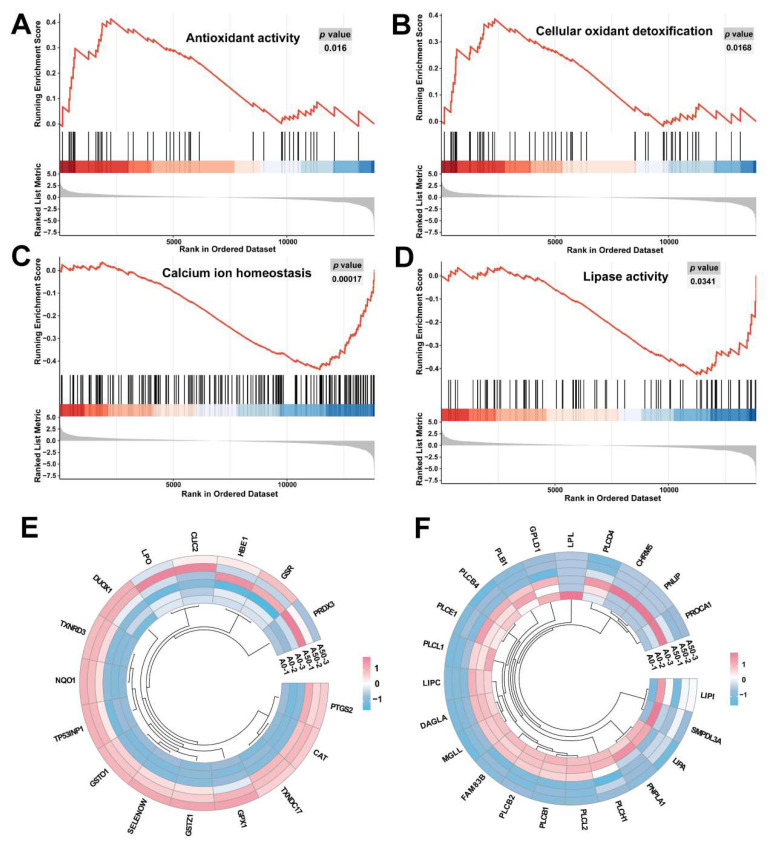
GSEA demonstrated that genes in AFB1 group were enriched in antioxidant activity (**A**), cellular oxidant detoxification (**B**), calcium ion homeostasis (**C**), and lipase activity (**D**). The Circos heatmaps revealed the association of the antioxidant activity (**E**) and lipase activity (**F**) related genes in AFB1 and control groups.

The gene set enrichment analysis (GSEA) enrichment plots (Figure 3A–D) displayed the up-regulation of pathways associated with antioxidant activity and cellular oxidant detoxification, while down-regulation was observed in the pathways related to calcium ion homeostasis and lipase activity. The Circos heatmaps visualize the expression of genes associated with antioxidant activity and lipase activity, as illustrated in Figure 3E and 3F, respectively. After 72 h of AFB1 treatment of SW480 cells, it was observed that the gene expression related to antioxidant activity and lipase activity exhibited an inverse alteration compared to the control group.

### 2.3. Proteomics Analysis Based on Differentially Expressed Proteins (DEPs)

**Figure 4:**
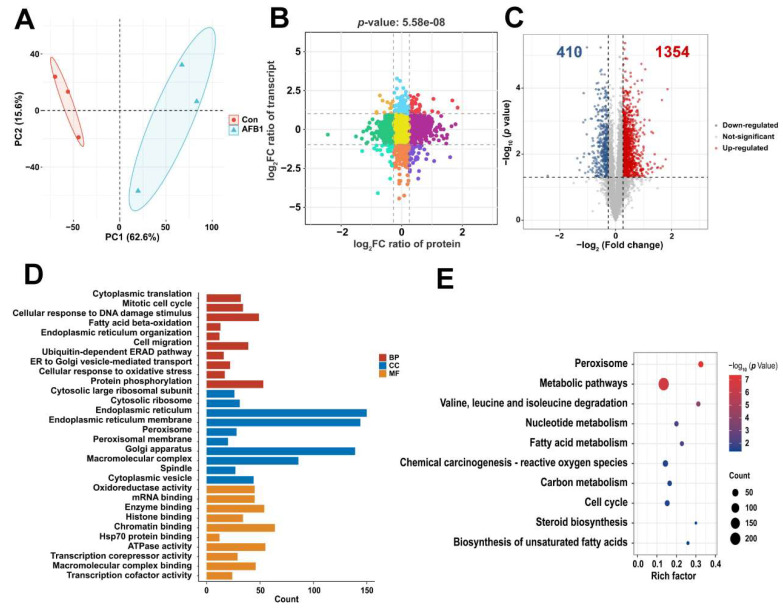
(**A**) The PCA of the protein expression between the AFB1 and control groups. (**B**) The nine-quadrant diagram depicting the correlation between protein expression and mRNA expression. (**C**) Volcano plot showcasing protein variations between AFB1 and the control group. Red represents up-regulated alterations, while blue represents down-regulated alterations. Analysis of enrichment of (**D**) GO terms (*p* < 0.05) and (**E**) KEGG pathway (*p* < 0.05) for DEPs.

**Figure 5:**
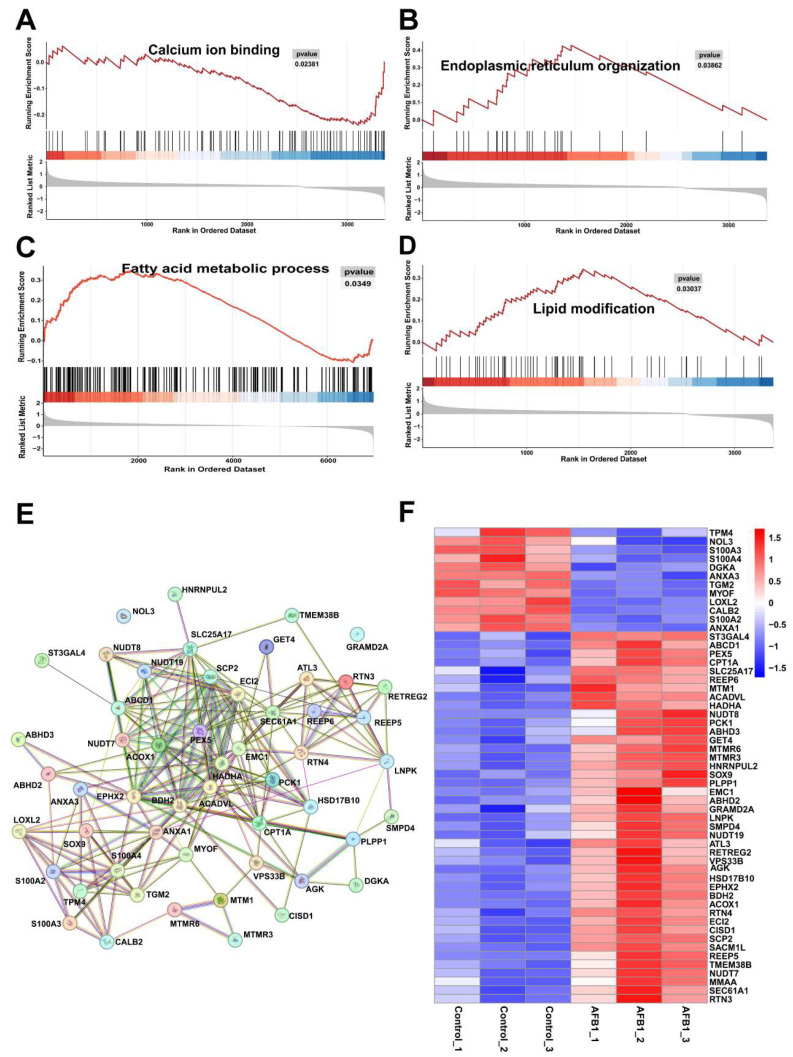
Comparison of the AFB1 group with the control group using GSEA. Calcium ion binding (**A**), endoplasmic reticulum organization (**B**), fatty acid metabolic process (**C**), and lipid modification (**D**). (**E**) The interaction among the genes related to calcium ion binding, endoplasmic reticulum organization, fatty acid metabolic process, and lipid modification. (**F**) The heatmap clearly demonstrates the significant disparity in the DEPs of calcium ion binding, endoplasmic reticulum organization, fatty acid metabolic process, and lipid modification.

The distinction in proteome samples between the untreated and treated AFB1 groups can be observed through the PCA score plots (Figure 4A). Correlation analysis of transcriptomic and proteomic results using nine quadrants revealed a total of 69 genes and proteins exhibiting concordant changes, with 41 showing up-regulation and 28 displaying down-regulation (Figure 4B). With *p* < 0.05 and a fold change >1.2 or <0.83 as the threshold, a total of 1764 DEPs were identified, including 1354 up-regulated proteins and 410 down-regulated proteins (Figure 4C). In order to further clarify the functional roles of the 1764 DEPs, GO and KEGG pathway enrichment analyses were conducted. The GO analysis demonstrated significant enrichment in fatty acid beta-oxidation, cellular response to oxidative stress, endoplasmic reticulum localization, and oxidoreductase activity (Figure 4D). The KEGG pathway is primarily enriched in metabolic pathways, fatty acid metabolism, and chemical carcinogenesis–reactive oxygen species (Figure 4E). GSEA enrichment analysis (Figure 5A–D) showed that calcium ion binding was down-regulated and endoplasmic reticulum organization, fatty acid metabolic process, and lipid modification were up-regulated. The PPI interaction network maps (Figure 5E) were constructed based on the proteins enriched in the aforementioned four pathways, and the corresponding protein expression levels were visualized in Figure 5F using heat maps. 

### 2.4. Analysis of the Metabolomics Data between the Group Treated with AFB1 and the Control Group

**Figure 6:**
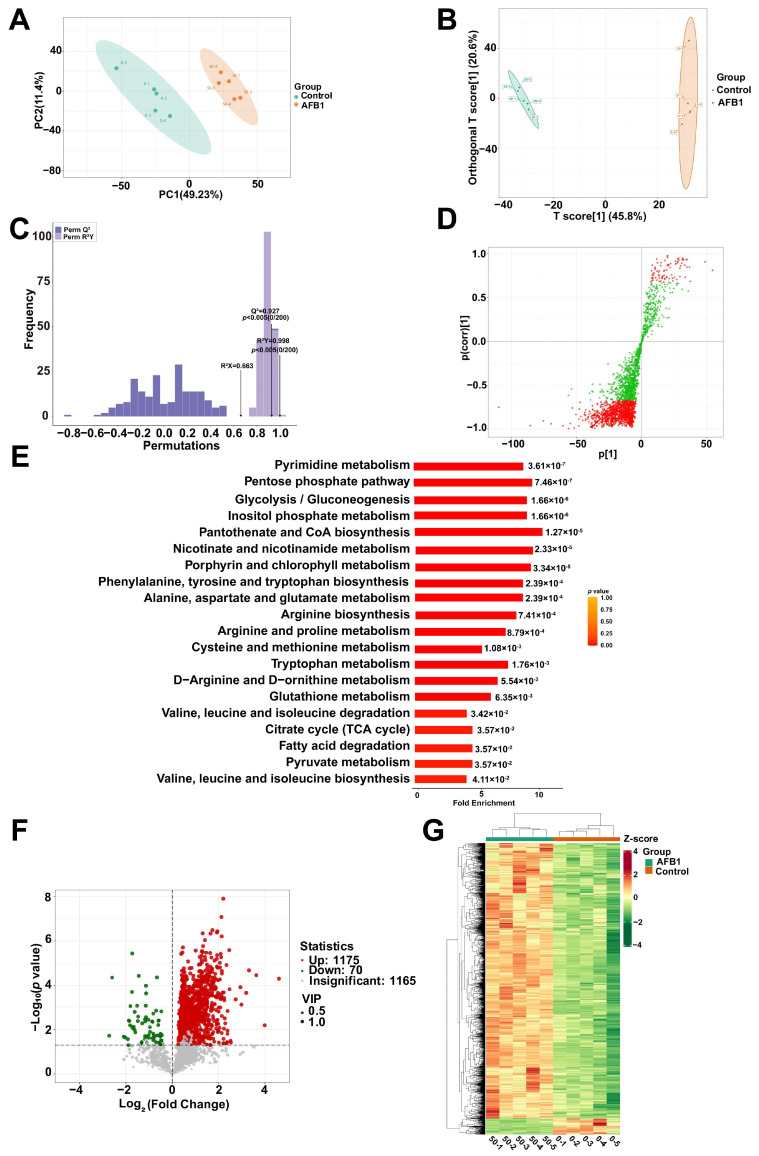
(**A**) The two-dimensional images resulting from the PCA analysis. (**B**) The OPLS-DA score plots indicate significant isolation and discrimination between the AFB1 and control groups. (**C**) The OPLS-DA model underwent testing with 200 random permutations. (**D**) OPLS-DA S-plot of the AFB1 and control groups. (**E**) MSEA enrichment analysis of DEMs between AFB1 and control groups. (**F**) The volcano plot shows DEMs between control groups and AFB1-treated group. (**G**) Heatmap of metabolomics between AFB1 and control groups. The DEMs with VIP scores > 1 and *p* value < 0.05 are displayed in the heatmap.

The PCA score scatter plots for all samples are presented in Figure 6A, revealing a marked distinction between the AFB1-treated and control groups. Further analysis of the data was carried out by means of orthogonal partial least squares discriminant analysis (OPLS-DA) (Figure 6B). The model-fitting parameter (R^2^X) and the model-discrimination parameter (R^2^Y) of the untargeted metabolomics approaches were, respectively, 0.663 and 0.998, suggesting that the model had a suitable fit (Figure 6C). The Q^2^ values in the original model were extremely close to 1, indicating the model’s reliability. In this study, 2410 metabolites were identified. The metabolites that have significant differences according to VIP values are shown in the OPLS-DA S-plot. (Figure 6D). The metabolite set enrichment analysis (MSEA) pathway analysis revealed that the metabolites were significantly enriched in pantothenate and CoA biosynthesis, glutathione metabolism, valine, leucine and isoleucine degradation, and fatty acid degradation (Figure 6E).

**Figure 7:**
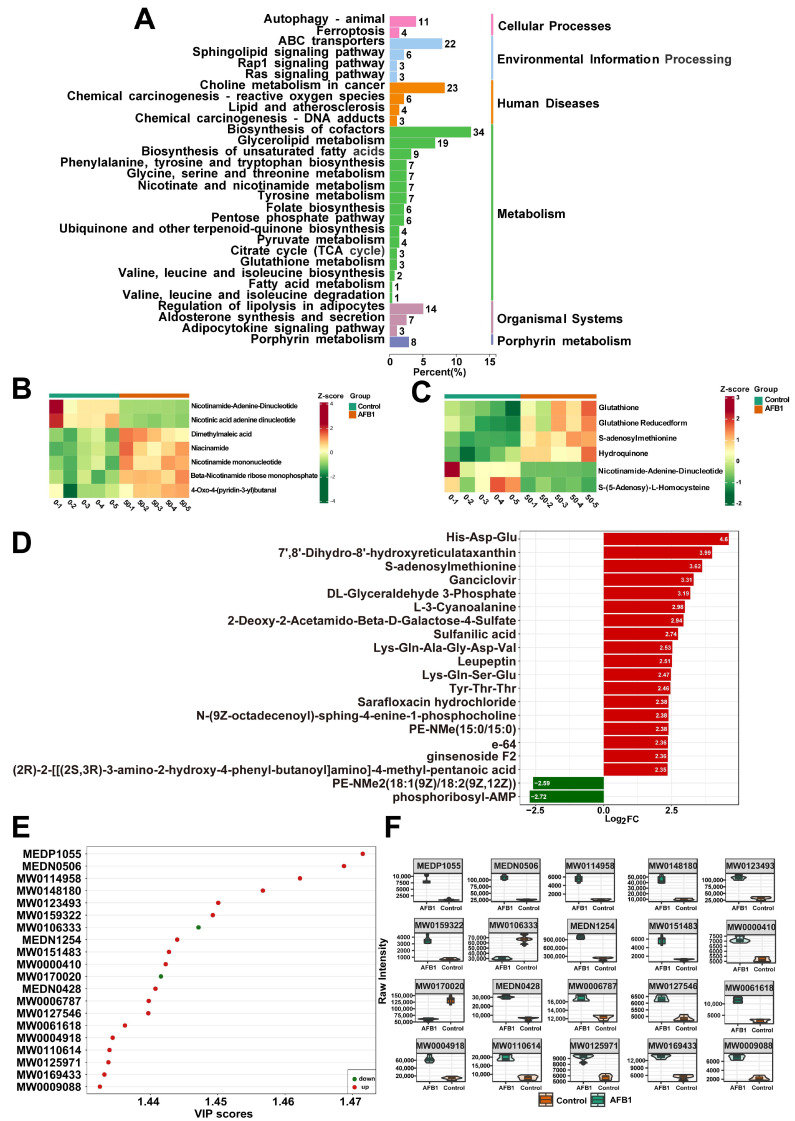
(**A**) The KEGG pathway analysis of DEMs between AFB1-treated and control groups. Heatmap of DEMs of nicotinic acid and nicotinamide metabolism (**B**) and chemical carcinogenesis–reactive oxygen species (**C**). (**D**) The bar plot shows representative metabolites that were significantly up-regulated or down-regulated in the top 20 DMEs. (**E**) The scatter plot of the top 20 DMEs for VIP values. (**F**) Metabolite violin diagram based on the top 20 VIP values.

Differential expression metabolites (DEMs) were defined as metabolites with VIP > 1 and *p* < 0.05. Subsequently, it was found that 70 metabolites were down-regulated, while 1175 metabolites were up-regulated (Figure 6F). As depicted in the volcano plot, the red and green dots represent up-regulated and down-regulated DEMs in SW480 cells, respectively. A heat map was constructed (Figure 6G) for the DEMs, which showed significant differences between the AFB1-treated and control groups. The DEMs detected were annotated using the KEGG database (Figure 7A). The KEGG classification results and enrichment analysis suggest that the DEMs revealed are related to cellular processes, human diseases, organismal systems, and environmental information processing. The enriched KEGG pathways include biosynthesis of unsaturated fatty acids, chemical carcinogenesis–DNA adducts, chemical carcinogenesis–reactive oxygen species, nicotinate and nicotinamide metabolism, porphyrin metabolism, lipid and atherosclerosis, and glutathione metabolism. Selection of DEMs in the chemical carcinogenesis–reactive oxygen species and nicotinate and nicotinamide metabolism pathways were made for cluster analysis (Figure 7B–C). A bar chart was constructed relying on the magnitude of the log2Fold change values for the DEMs (Figure 7D). In the AFB1-treated group, the most remarkable up-regulation compared to the control group was witnessed in His–Asp–Glu, 7′,8′-Dihydro-8′-hydroxyreticulataxanthin, and the S-adenosylmethionine metabolite. In the OPLS-DA model, the top 20 DEMs having the most significant VIP values were chosen to construct a scatter plot (Figure 7E) and violin plot (Figure 7F) to show the differences and changes in metabolites more lucidly and visually. The names of the 20 metabolites are listed in Table S1 corresponding to the IDs.

### 2.5. The Potential Target for AFB1 Toxicity

**Figure 8:**
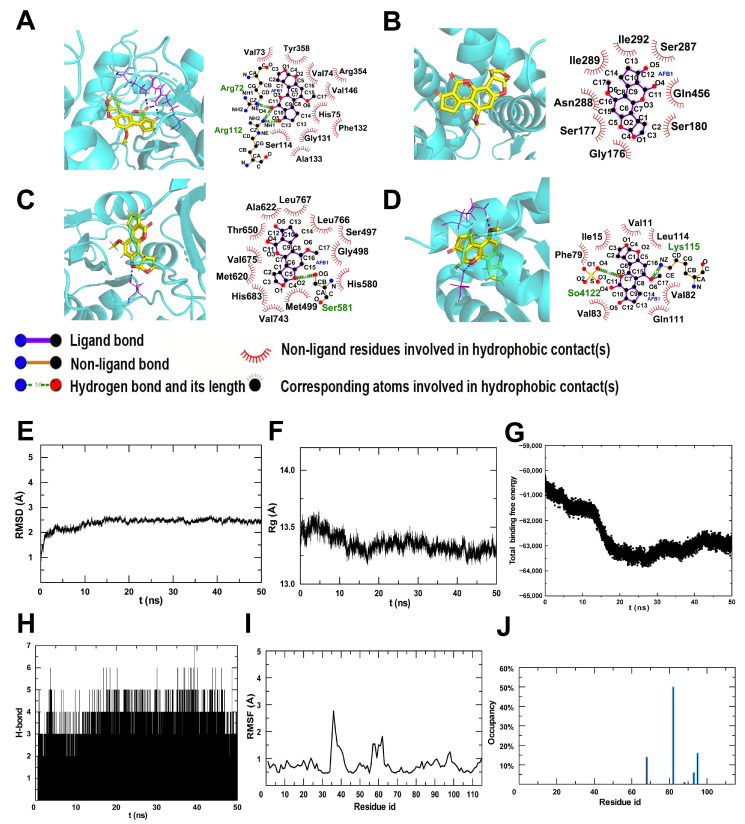
The interactions between AFB1 and related proteins of CAT (**A**), SEC61A1 (**B**), ACSL1 (**C**), and SCP2 (**D**). (**E**) Time evolution of RMSD, Rg (**F**), total binding free energy (**G**), and hydrogen bond number (**H**). (**I**) RMSF of each residue from SCP2. (**J**) H-bond occupancy rate of each residue.

It can be seen from Table 1 and Figure 8A–D that the interactions between AFB1 and related proteins were predicted. Specifically, AFB1 bound CAT through the formation of three hydrogen bonds at Arg72 and Arg112, as well as ten hydrophobic contacts with Val73, Tyr358, Val74, Arg354, Val146, His75, Phe132, Gly131, Ser114, and Ala133 (Figure 8A). AFB1 bound ACSL1 by forming one hydrogen bond at Ser581 and twelve hydrophobic contacts with Met620, His683, Val743, Met499, Thr650, Val675, Ala622, Leu767, His580, Ser497, Leu766, and Gly498 (Figure 8B). Additionally, AFB1 bound SCP2 through the formation of two hydrogen bonds at So4122 and Lys115, along with seven hydrophobic contacts with Leu114, Val82, Gln111, Val83, Phe79, Ile15, and Val11 (Figure 8C). Finally, AFB1 bound SEC61A1 by forming seven hydrophobic contacts with TIle292, Ser287, Gln456, Ser180, Gly176, Ser177, and Asn288 (Figure 8D). The protein SCP2 plays a crucial role in facilitating the redistribution of cholesterol from the plasma membrane to various intracellular locations, and promoting efficient cholesterol transport from the plasma membrane to the endoplasmic reticulum [23]. In Figure 8E–H, the time evolution of the root mean square deviation (RMSD), radius of gyration (Rg), total binding free energy, and hydrogen bond number are shown. The binding of the AFB1-SCP2 complex remained stable throughout the 50 ns dynamic simulation, with RMSD and root mean square fluctuation (RMSF) values (Figure 8I) fluctuating by less than 5Å. The hydrogen bond occupancy rate of each residue is depicted in Figure 8J, with three sites exhibiting an occupancy rate exceeding 10%. The occupancy rate of the hydrogen bond involving isoleucine in complex 82 exhibited the highest value, reaching 50%.

### 2.6. Oxidative Stress Induced by AFB1

**Figure 9:**
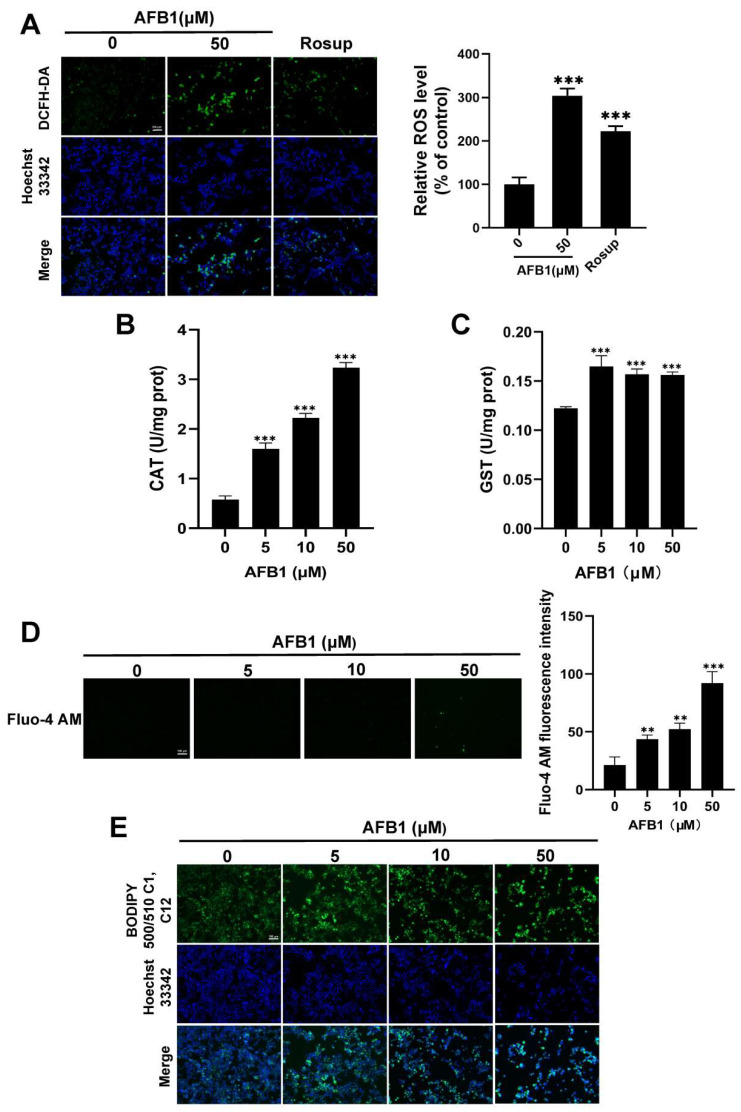
(**A**) Intracellular ROS were detected by DCFH-DA in fluorescence microscopy images. The CAT (**B**) and GST (**C**) enzyme activity. (**D**) Cytoplasmic Ca^2+^ imaging of SW480 cells. (**E**) BODIPY 500/510 C1, C12 probe was used to detect fatty acid in SW480 cells. Scale bar = 100 μm. ** *p* < 0.01, *** *p* < 0.001, n = 3.

Intracellular reactive oxygen species (ROS) levels in SW480 cells were assessed using the fluorescent probe DCFH-DA. A significant increase in ROS levels was observed when cells were exposed to 50 μM AFB1 compared to the control group (Figure 9A). The fluorescence intensity of DCFH-DA showed a significant increase from 100% in the control group to 304% in the group treated with 50 µM AFB1. Overall, AFB1 stimulation triggered the production of intracellular ROS in SW480 cells.

### 2.7. The Antioxidant Enzyme Activity

Figure 9B–C demonstrate a significant increase in CAT and GST levels in AFB1-treated SW480 cells compared to untreated cells. The quantitative values of CAT activity were 0.58 ± 0.07, 1.60 ± 0.12, 2.22 ± 0.09, and 3.24 ± 0.1 U/mg prot in the 0, 5, 10, and 50 μM AFB1 groups, respectively. The quantitative values of GST activity were 0.122 ± 0.002, 0.165 ± 0.011, 0.157 ± 0.005, and 0.156 ± 0.003 U/mg prot, respectively. These results imply an imbalance in the cellular oxidative and antioxidant levels, leading to the occurrence of oxidative stress.

### 2.8. The Exposure to AFB1 Leads to Excessive Accumulation of Intracellular Ca^2+^

The representative images depicting intracellular Ca^2+^ levels are presented in Figure 9D, illustrating the dose-dependent impact of AFB1 on Ca^2+^ concentrations in SW480 cells. The *p* values for the 0, 5, 10, and 50 μM AFB1 groups were found to be 0.004, 0.001, and 0.000001, respectively. The green fluorescence results revealed the occurrence of cellular Ca^2+^ overload.

### 2.9. Fatty Acid Localization

BODIPY 500/510 C1, C12 is a BODIPY fluorescent-labeled saturated fatty acid capable of traversing the cellular membrane to specifically stain polar lipids within the cell. When the probe is incorporated into living cells, an excitation dimer can be formed with a maximum excitation wavelength of 500 nm and a maximum emission wavelength of 510 nm. As shown in Figure 9E, the green fluorescence indicated the condensation of lipid droplets after AFB1 treatment, and the control group was labeled with a normal cell membrane. The result indicates that the transport and metabolism of fatty acids in SW480 cells exhibit impairments.

### 2.10. AFB1-Induced Mitochondrial Dysfunction

**Figure 10:**
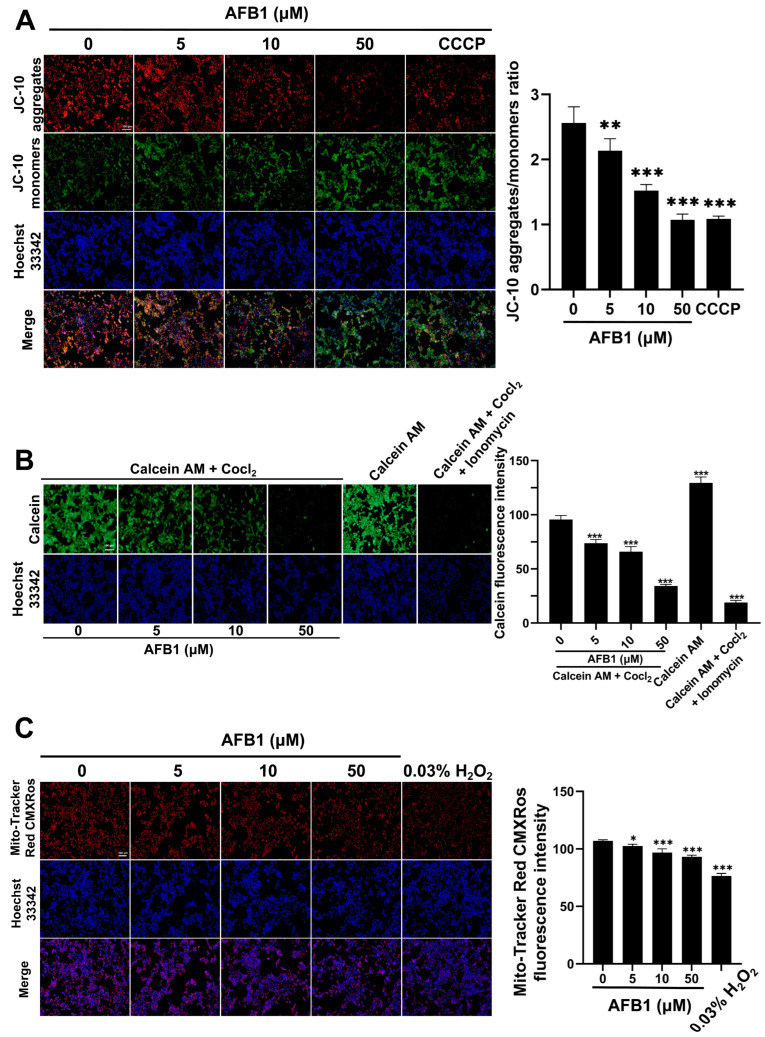
(**A**) JC-10 probe was used to detect the MMP in SW480 cells. (**B**) Representative fluorescence image and quantitative results of mPTP detection in SW480 cells using calcein AM probe after 72 h of AFB1 treatment. (**C**) The MitoTracker Red CMXRos probe was used to detect mitochondrial activity in SW480 cells. Scale bar = 100 μm. * *p* < 0.05, ** *p* < 0.01, *** *p* < 0.001, n = 3.

In comparison with the control group, the red–green fluorescence ratio in the groups treated with 0, 5, 10, and 50 μM of AFB1 exhibited a downward trend, with values of 2.14 ± 0.18, 1.52 ± 0.09, and 1.07 ± 0.09, respectively (Figure 10A). This demonstrated that treating with AFB1 caused a decrease in the MMP of SW480 cells. The assessment of mitochondrial function was conducted using the mPTP. The mPTP is a non-specific channel composed of the inner and outer mitochondrial membranes which serves as the structural basis for mitochondrial function. Under physiological conditions, the mPTP toggles between the open and closed states with the uptake and release of Ca^2+^ by mitochondria. However, in the event of oxidative damage or cell death, the mPTP would remain continuously open. 

The Calcein AM probe can passively enter cells and accumulate in mitochondria, where the nearly nonfluorescent Calcein AM is hydrolyzed by esterase to remove the acetyl methyl ester, resulting in the formation of the polar fluorescent dye calcein and the emission of intense green fluorescence. Additionally, when the opening degree of the mPTP increases, Co^2+^ can enter the mitochondria and form a complex with calcein, causing the quenching of the green fluorescence signal. Therefore, we can determine the opening degree of the mPTP based on the intensity of the green fluorescence. Ionomycin is a Ca^2+^ ionophore that induces the influx of extracellular Ca^2+^ into the mitochondrial matrix, leading to the opening of the mPTP, and is commonly used as a positive control. In Figure 10B, the Calcein AM and Calcein AM + Ionomycin + Cocl_2_ groups are the negative and positive controls, respectively. The fluorescence intensities of the calcein were measured as 95.51 ± 3.84, 73.64 ± 3.60, 65.89 ± 4.69, and 34.23 ± 1.47 in the 0, 5, 10, and 50 μM AFB1 groups (*p* < 0.001), respectively. The significant decrease in calcein fluorescence intensity indicated that AFB1 over-activated the opening of the mPTP, causing mitochondrial damage in the SW480 cells. After AFB1 treatment, the fluorescence intensity results of MitoTracker Red CMXRos indicated that the mitochondrial activity of the SW480 cells decreased (Figure 10C).

### 2.11. mRNA and Protein Expression Involved in Oxidative Stress and Lipid Signaling Pathways

**Figure 11:**
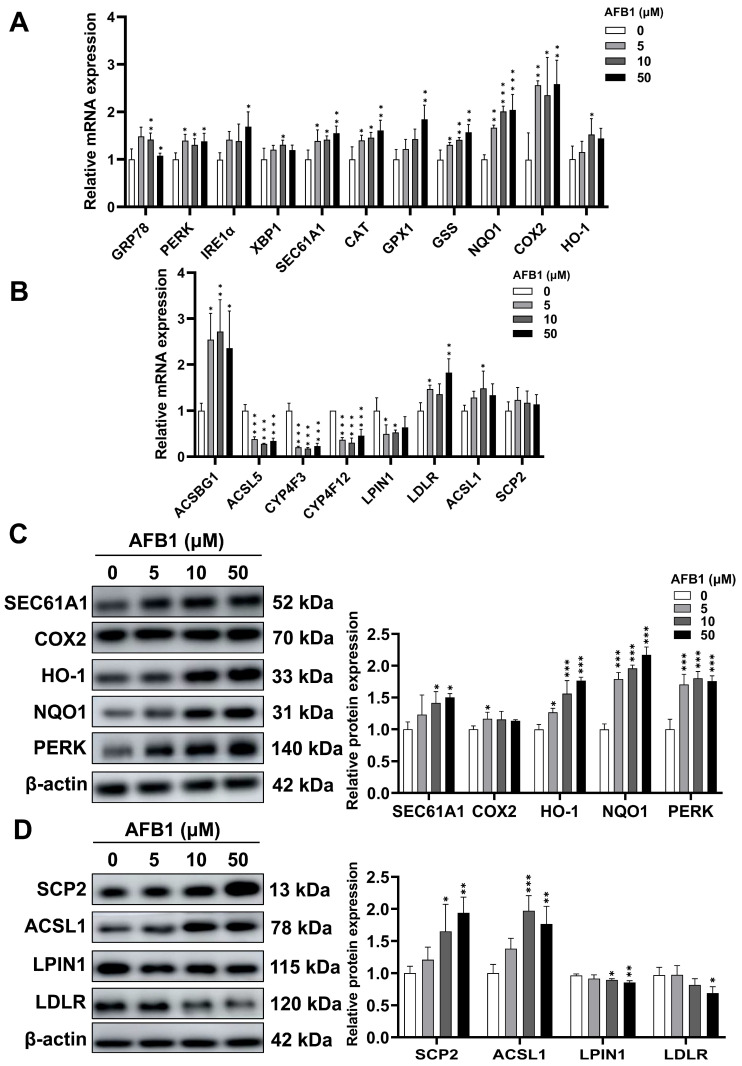
The levels of mRNA were assessed via RT-qPCR analysis pertaining to oxidative stress (**A**) and lipid metabolism (**B**). (**C**) The protein expression levels of SEC61A1, COX2, HO-1, NQO1, and PERK in 0, 5, 10, and 50 μM AFB1-treated SW480 cells. (**D**) The protein expression levels of SCP2, ACSL1, LPIN1 and LDLR in 0, 5, 10, and 50 μM AFB1-treated SW480 cells. * *p* < 0.05, ** *p* < 0.01, and *** *p* < 0.001, n = 3.

The toxicity effect of AFB1 on the intestine was assessed by examining 19 genes associated with oxidative stress and lipid signaling pathways. Compared to the control group, the *GRP78*, *PERK*, *IRE1*α, *XBP1*, *SEC61A1*, *CAT*, *GPX1*, *GSS*, *NQO1*, *COX2*, *HO-1* genes related in the oxidative stress pathway were up-regulated (Figure 11A). In the lipid synthesis and fatty acid metabolism pathways, except for the up-regulated expression of *ACSBG1*, *ACSL1*, *LDLR*, and *SCP2* genes, the *ACSL5*, *LPIN1*, *CYP4F3*, *CYP4F12* genes were down-regulated (Figure 11B). Figure 11C–D show that the results of western blotting analysis indicate a considerable enhancement in the expression levels of SEC61A1, COX2, HO-1, NQO1, PERK, SCP2, and ACSL1 in the AFB1 group as opposed to the control group. Furthermore, the expression levels of LPIN1 and LDLR also dropped in a concentration-dependent manner in response to AFB1. 

## 3. Discussion

The intestinal tract functions as a crucial barrier against toxins and detrimental substances. Although AFB1 has been demonstrated to possess hepatotoxic properties, it can also induce intestinal damage both in vivo and in vitro [24,25]. Histological examination revealed that the intestinal inflammation triggered by the pathogenic dosage of AFB1 was less than that in the liver [26]. Furthermore, colon injury was observed following low-dose AFB1 treatment prior to any discernible signs of liver damage. 

With the development of high-throughput sequencing and microarray technologies, researchers were allowed to monitor the expression levels of thousands of genes across a genome simultaneously. Wu et al. [27] showed the m6A methylome and transcriptome profiles in HCT116 cells treated with AFB1, revealing that AFB1-induced HCT116 cytotoxicity is associated with perturbations in the levels of m6A methylation modifications and abnormal regulation of m6A regulators. Transcriptomic analysis was employed to elucidate the mechanism underlying afb1 toxicity in aquatic environments and organisms. In a study investigating the hepatotoxicity of AFB1 in developing juvenile zebrafish, transcriptome sequencing analysis revealed that AFB1 perturbed liver redox levels, thereby inducing oxidative stress, steatosis, and lipid droplet accumulation [28]. In our study, the 1037 DEGs in SW480 cells treated with AFB1 were enriched in pathways related to calcium, triglyceride lipase activity, long-chain fatty acid metabolic processes, and responses to oxidative stress. The genes *CAT* and *NQO1* were found to be enriched in response to oxidative stress, as evidenced by the significant increase in their levels observed in the IGV diagram. The DEGs identified through RT-qPCR were consistent with the sequencing results, thereby validating the findings of transcriptome analysis. These findings suggest an imbalance between oxidation and antioxidant levels within the cell, leading to oxidative stress occurrence. These findings provide a theoretical basis for understanding the mechanisms of AFB1 toxicity and present new strategies for the prevention, diagnosis, and treatment of AFB1 toxicity.

According to the genetic central dogma, genetic information encoded in the DNA sequence of a gene is transcribed into mRNA, which subsequently undergoes translation to synthesize proteins and execute diverse cellular functions. Proteomics is an effective tool for comprehensively understanding genome expression, and can complement the study of genome modification and translation. Multi-omics strategies, such as combining epitranscriptomics, proteomics, and metabolomics, help to reveal new mechanisms and identify new targets. In the previous study, through the multi-omics combined analysis of miRNA sequencing, transcriptomics, and proteomics, it was found that AFB1 and AFM1 exert an additive toxic effect on NCM460 cells via the p53 signaling pathway [29]. In our study, proteomic data showed that after the treatment of SW480 cells with AFB1, the regulation of terms, such as endoplasmic reticulum organization, fatty acid metabolic process, lipid modification, and calcium ion binding, were altered. Furthermore, the key proteins related to the terms have been discovered in previous studies to be associated with intestinal lipid metabolism. The protein SCP2, which is differentially expressed and enriched by the proteome in this study, has been demonstrated to facilitate the translocation of cytoplasmic lipid peroxidation (LPO) into mitochondria during RSL3-induced ferroptosis in chondrocytes. This process leads to mitochondrial membrane impairment and the release of ROS [30]. Among them, the up-regulation of SCP2 led to changes in pathways involved in lipid uptake and absorption in zebrafish of intestinal epithelial cells [31]. These indicated that the involvement of SCP2 in AFB1-induced cytotoxicity may represent a significant target worthy of investigation.

Metabolomics, a frequently utilized approach for uncovering metabolic alterations induced by external stimuli, has found extensive application in diverse research domains such as toxicology, drug development, and environmental science [32,33,34]. Metabolites are the products of gene–environment interactions and have a close association with disease phenotypes [35]. In the present study, metabolomics detected 2410 metabolites, and MSEA results indicated their association with glutathione metabolism and fatty acid degradation. Glutathione is the most abundant soluble sulfhydryl antioxidant in cells, which plays an important role in antioxidant defense, protein function regulation, gene expression, and cell proliferation [36]. Fatty acids are structural components of cell membrane phospholipids, able to esterify with glycerol or sterol backbones to generate triacylglycerols or sterol esters, respectively, and then kept in lipid droplets [37,38]. Therefore, the significant changes in glutathione metabolism and fatty acid degradation caused by AFB1 treatment might have led to a redox imbalance and lipid metabolism disorder in SW480 cells, resulting in decreased cell viability and mitochondrial damage. 

The validation in this study was limited to a single cell line SW480 cells, which are cancer cells with distinct metabolic and physiological states compared to normal cells. This limits the ability to further elucidate the potential mechanism of AFB1-induced cytotoxicity. Future research will include the establishment of animal and organoid models for rats and mice, as well as the isolation of human primary intestinal epithelial cells, in order to investigate the toxic mechanism of AFB1 in the intestinal tract. 

## 4. Conclusions

This study demonstrated the toxic effects of AFB1 on SW480 cells. Multi-omics analysis revealed that oxidative stress and lipid metabolism may be involved in AFB1-mediated cytotoxicity. According to the experimental results, AFB1 can reduce cellular viability, increase ROS and calcium ions, decrease mitochondrial activity, lead to fatty acid metabolism disorders, and eventually lead to mitochondrial damage and lipid metabolism disorders. The aforementioned findings serve as a crucial foundation for elucidating the cytotoxicity of AFB1.

## 5. Materials and Methods

### 5.1. Chemical Reagent and Cell Culture

The chemical formula of AFB1 is C_17_H_12_O_6_, and its chemical structure is depicted in Figure 1A. AFB1 was obtained from Macklin (Shanghai, China), with a purity level of 98.275%. The human SW480 cells utilized in this study were cultured in a constant temperature incubator at 37 °C using Dulbecco’s Modified Eagle medium supplemented with 10% fetal bovine serum. The AFB1 compound was dissolved in DMSO, with subsequent experiments conducted using working concentrations of 5, 10, 50, and 100 μM.

### 5.2. Cell Viability Assay

The MTT cytotoxicity assay was utilized to evaluate the viability of SW480 cells that were exposed to AFB1 [39]. SW480 cells were seeded in 96-well plates and treated with 0, 5, 10, 50, and 100 μM AFB1 for 72 h. We prepared a 5 mg/mL MTT solution and added 10 μL of the solution along with 90 μL of the medium to each well at 37  °C with 5% CO_2_. After a duration of 4 h, the OD at 490 nm was quantified for each well subsequent to the addition of DMSO.

### 5.3. RNA Extraction and Transcriptomics

The SW480 cells were seeded in 6 cm dishes with a density of 3 × 10^6^ cells per dish. The RNA was extracted from SW480 cells cultured with 0 and 50 μM AFB1 for 72 h using the Trizol reagent (Invitrogen, Carlsbad, CA, USA) method. The mRNA sequencing was conducted by Seqhealth Technology Co., Ltd. (Wuhan, China) on triplicate RNA samples for each group. Briefly, the polyadenylated RNA enrichment was performed using VAHTS mRNA capture beads with a total of 10 μg of RNA, while the resulting mRNA fragments were predominantly distributed within the range of 100–200 nt. The sequencing library was constructed using the KC-Digital stranded mRNA library prep kit (seqhealth). Sequencing of the final libraries was carried out on the Novaseq 6000 (Illumina) with a read length of 200–500 bps. The sequencing mRNA data were qualified twice. HISAT2 was used for comparison against the human genome, and the resulting count number was applied to identify DEGs with the aid of the DeSeq2 package.

### 5.4. Tandem Mass Tag Proteomics Analysis

The SW480 cells were exposed to 0 and 50 μM AFB1 for 72 h, followed by the addition of a lysate buffer containing 100 mM triethylammonium bicarbonate and 1% SDS. The protein was reduced using Tris(2-carboxyethyl) phosphine hydrochloride and subsequently alkylated with iodoacetamide. This was followed by precipitation with six volumes of ice-cold acetone and subsequent blow-drying. After resuspending in a triethylammonium bicarbonate buffer, enzymolysis was performed using trypsin and incubated at 37 °C for 20 h. A set of TMTpro isobaric tags were utilized to tag the peptides, and the labeling reaction was carried out at ambient temperature for 60 min. The High-pH Reversed-Phase Peptide Fractionation Kit (Invitrogen) was employed to fractionate the labeled peptides into 15 fractions. The separated peptides were analyzed using the EASY-nLC 1200 UHPLC System and Orbitrap Exploris 480 MS for combined detection. The mass spectrometry files were processed using Proteome Discoverer version 3.0, and the statistical analysis of the expression matrix was conducted utilizing RStudio (2022.12.0-353).

### 5.5. Sample Preparation and Analysis for Metabolomics

SW480 cells treated with AFB1 for 72 h were washed with PBS. Into the cell sample, a 500 μL solution containing the internal standard was added and vortexed to suspend the sample. The sample was frozen rapidly by immersing it in liquid nitrogen for 5 min and then thawed in dry ice and ice for 5 min, respectively. Then, the solution was vortexed for 2 min to ensure thorough mixing, and this process was repeated three times. The centrifugation of the samples was performed at 12,000 r/min for 10 min at 4 °C. The supernatant was placed in −20 °C for 30 min and underwent centrifugation at 12,000 r/min for 3 min at 4 °C, followed by collecting the supernatant for LC-MS analysis. A 2.1 mm × 100 mm ACQUITY UPLC HSS T3 C18 column from Waters (Milford, MA, USA) was employed for the purpose of elution. After gradient elution, the column temperature was set to 40 °C, accompanied by an injection volume of 4 μL and a flow rate of 0.4 mL/min. Finally, the metabolomics data were processed and analyzed. 

### 5.6. Molecular Docking

Molecular docking was completed using SYBYL-X 2.0 software, after the 3D conformations of proteins were obtained from the Protein Data Bank [40]. Water molecules and heteroatoms of protein conformation were removed, and hydrogen atoms were added for preprocessing using SYBYL-X 2.0. The 2D structure of AFB1 was retrieved PubChem and imported into Open Babel GUI 2.4.1 to generate the mol2 format. Semi-flexible docking was utilized for the docking of proteins with AFB1. The interaction between the protein and the molecule was regarded as stable when the score value exceeded 5.

### 5.7. Molecular Dynamics (MD)

The MD simulations and calculations were performed using the SPONGE 1.4 and XPONGE2 software packages [41]. The ff14SB united-atom force field3 was employed throughout the simulation study. By using the SPC/E water model in cubic boxes, the ligand–protein complex was solved, ensuring that the boxes were filled with water and that there was a minimum distance of 1.2 nm from each side of the cube. Prior to the formal dynamics simulation, preliminary NVT and NPT simulations were conducted for a duration of 100 ps. The system underwent a 50 ns dynamic simulation, with the temperature held at 300 K and the atmospheric pressure set to 1 bar.

### 5.8. ROS Measurement by DCFH-DA

To investigate the effects of AFB1 on oxidative stress in SW480 cells and measure ROS levels, the SW480 cells treated with 50 µM AFB1 for 72 h were incubated with 1 µM DCFH-DA probe (Beyotime, Shanghai, China) at a temperature of 37 °C for 40 min. After washing the stained cells with PBS, we observed them using a fluorescence microscope (Zeiss, Oberkochen, Germany) and fluorescence intensity was quantitatively detected using ImageJ software (version 1.54d), and the mean gray value, defined as the Integrated Density divided by area, was taken as the detection result.

### 5.9. Assay of Catalase (CAT) and Glutathione S-Transferase (GST)

SW480 cells in 6-well plates were scraped and collected after a 72 h treatment with AFB1 at concentrations of 0, 5, 10, and 50 μM. The reagent provided in the kit was added to the cell precipitate, followed by placing the cells on ice for ultrasonication. The subsequent suspension was subjected to centrifugation at 8000× *g* and 4 °C for a duration of 10 min. The supernatant was utilized for the quantification of CAT and GST activities, with absorbance measurements performed at 405 nm and 340 nm as per the provided instructions, respectively.

### 5.10. Measurement of Cellular Calcium

The cellular calcium detection was performed in SW480 cells following a 72 h incubation with treatments of 0, 5, 10, and 50 μM AFB1. The calcium concentration was assessed by quantifying the fluorescence intensity of Fluo-4 AM dye (Beyotime) loaded in the cytoplasm. The cell samples were incubated at 37 °C for 40 min with a diluted Fluo-4 AM to a final concentration of 1 μM in PBS. The variations in green fluorescence at 494 and 516 nm excitation and emission wavelengths were captured by a fluorescence microscope (Zeiss).

### 5.11. Fatty Acid Green Fluorescence Probe

The BODIPY 500/510 C1, C12 fatty acid green fluorescence probe was employed in this study to investigate lipid accumulation. Cells treated with 0, 5, 10, 50 μM AFB1 for 72 h in 24-well plates were incubated with 5 μM BODIPY 500/510 C1, C12 for 15 min. After washing with PBS, the green fluorescence with an emission wavelength of 510 nm was observed by fluorescence microscope (Zeiss).

### 5.12. Detection of Mitochondrial Membrane Potential (MMP)

MMP was assessed by making use of the JC-10 probe, which was obtained from Solarbio (Beijing, China). Generally, MMP is maintained at a relatively high level. In the mitochondrial matrix, the JC-10 probe forms aggregates and emits red fluorescence. However, when mitochondria are damaged, JC-10 exists as monomers and results in green fluorescence. SW480 cells were treated to 0, 5, 10, and 50 μM of AFB1 for 72 h, followed by incubation with the JC-10 probe and Hoechst 33342 at 37 °C in the dark for 20 min and 10 min, respectively. Finally, fluorescent images are obtained with the help of a microscope (Zeiss).

### 5.13. Detection of Mitochondrial Permeability Transition Pore (mPTP)-Opening

To assess the impact of AFB1 on mitochondrial function in SW480 cells, we utilized the Calcein AM probe (Beyotime) to detect the opening of the mPTP. Briefly, SW480 cells were seeded into 24-well plates at 8 × 10^4^ cells per well and treated with 0, 5, 10, and 50 μM AFB1 for 72 h. Subsequently, 250 μL of Calcein AM staining solution was added to each well and incubated at 37 °C for 40 min. Following that, the culture medium was added to replace the Calcein AM staining solution and further incubated for 20 min. Finally, the cell fluorescence was evaluated using a fluorescence microscope (Zeiss) and then the Image J software was employed for the quantitative analysis.

### 5.14. Assessment of Mitochondrial Activity

Mitochondrial activity was measured using the MitoTracker Red CMXRos probe (Beyotime), which specifically tags bioactive mitochondria and fluoresces in red. SW480 cells were treated with 0, 5, 10, and 50 μM of AFB1 for 72 h, followed by incubation with the MitoTracker Red CMXRos probe and Hoechst 33342 at 37 °C in the absence of light for 30 min and 10 min, respectively. Finally, a fluorescence microscope (Zeiss) was used to observe the fluorescence intensity of SW480 cells.

### 5.15. RT-qPCR

The RNA-extraction procedure is consistent with the previously described methodology. According to the directions of the Evo M-MLV RT Reaction Mix kit (Accurate Biology, Changsha, China), the total RNA was reverse-transcribed into a cDNA template. The primer sequences of the selected genes utilized in this investigation are presented in Table 2. RT-qPCR was implemented by means of the AtiaMX real-time PCR system from Agilent (Santa Clara, CA, USA). The PCR comprised an initial denaturation step at 95 °C for 30 s, followed by 40 cycles at 95 °C for 5 s, and 60 °C for 30 s. The dissociation curve is obtained through the dissolution procedure specified by the machine’s default settings. By using the 2^−∆∆CT^ method [27], the relative mRNA level of each targeted gene was standardized to *β-actin*.

### 5.16. Western Blotting

Using RIPA buffer (Beyotime), SW480 cells were lysed to extract protein after being treated with 0, 5, 10, and 50 μM AFB1 for 72 h. Proteins were fractionated on 10% sodium dodecyl sulfate–polyacrylamide gel electrophoresis and subsequently transferred to a PVDF membrane. The bands were blocked with 5% non-fat milk powder for 1.5 h. Primary antibodies were added to the membranes and incubated overnight at 4 °C [42]. The antibodies utilized were SEC61A1 (ABclonal, Wuhan, China), COX2 (Abclonal), HO-1 (Proteintech, Wuhan, China), NQO1 (Proteintech), PERK (Proteintech), SCP2 (Proteintech), ACSL1 (Proteintech), LPIN1 (Proteintech), LDLR (Proteintech), and β-actin (Proteintech), which were used at a dilution of 1:1000, 1:1000, 1:3000, 1:4000, 1:1000, 1:2000, 1:3000, 1:5000, 1:2000, and 1:2500, respectively. Then, peroxidase-conjugated goat anti-rabbit IgG secondary antibodies (1:10,000, Proteintech) were incubated with the membranes for 90 min. Exposure was carried out via the Amersham Imager 680 from GE Healthcare Bio-Sciences AB (Uppsala, Sweden), followed by the utilization of the Image J software for the quantification of the target bands.

### 5.17. Statistical Analysis

All statistical data were analyzed by using SPSS 26 (IBM, New York, NY, USA). One-way ANOVA was utilized for group differences and presented as the mean ± standard deviation (SD). A *p* value less than 0.05 was considered statistically significant.

## Figures and Tables

**Table 1:** Interactions between AFB1 and oxidation and lipid-related proteins.

Protein	PDB ID	Total Score	Crash	Polar	H-Bond Number	Residues Involved in H-Bond Formation	Hydrophobic Contact Number	Residues Involved in Hydrophobic Contacts
CAT	8HID	7.7967	−0.2167	0	3	Arg72, Arg112 (2 H-bonds)	10	Val73, Tyr358, Val74, Arg354, Val146, His75, Phe132, Gly131, Ser114, Ala133
SEC61A1	8DNV	5.6242	−0.7089	2.9825	0	−	7	Ile292, Ser287, Gln456, Ser180, Gly176, Ser177, Asn288
ACSL1	2JFK	5.5354	−0.9486	1.9938	1	Ser581	12	Met620, His683, Val743, Met499, Thr650, Val675, Ala622, Leu767, His580, Ser497, Leu766, Gly498
SCP2	1IKT	5.1034	−0.5708	1.8757	2	So4122, Lys115,	7	Leu114, Val82, Gln111, Val83, Phe79, Ile15, Val11
LPIN1	5WGB	4.8557	−0.6691	3.1075	2	Tyr26, Arg34	5	Val33, Thr415, Glu417, Thr30, Glu416
NQO1	5EA2	4.24	−0.6254	1.0619	2	Lys113, Tyr126	4	Tyr67, Phe120, Glu117, Phe178

**Table 2:** Sequences of primers used in RT-qPCR.

Gene	Forward Primer (5′-3′)	Reverse Primer (5′-3′)
*GRP78*	CAGTTGTTACTGTACCAGCCTA (F)	CATTTAGGCCAGCAATAGTTCC (R)
*PERK*	CCAGTTTTGTACTCCAATTGCA (F)	CAGATACAGCTGGCCTCTATAC (R)
*IRE1α*	CGTGAGCGACAGAATAGAAAAG (F)	GCTTCTTATTTCTCATGGCTCG (R)
*XBP1*	CTTGTAGTTGAGAACCAGGAGT (F)	CCCAACAGGATATCAGACTCTG (R)
*SEC61A1*	AATTTGAAGGTGCTATCATCGC (F)	ACGTATAGAAGAGCTTGATGGG (R)
*CAT*	GAGCACAGCATCCAATATTCTG (F)	CTCATTCAGCACGTTCACATAG (R)
*GPX1*	GTTGCCTGGAACTTTGAGAAG (F)	CTCGATGTCAATGGTCTGGAAGG (R)
*GSS*	CTCTTTGACATCCACAAGCAAG (F)	AACATGTAGTCTGAGCGATTCA (R)
*NQO1*	CAACCACGAGCCCAGCCAATC (F)	ACTCCACCACCTCCCATCCTTTC (R)
*COX2*	TGTCAAAACCGAGGTGTATGTA (F)	AACGTTCCAAAATCCCTTGAAG (R)
*HO-1*	CCTCCCTGTACCACATCTATGT (F)	GCTCTTCTGGGAAGTAGACAG (R)
*ACSBG1*	AAACAAGATGGCCAATGTGTAC (F)	CGTGATATTGTCTTGACTCAGC (R)
*ACSL5*	ATATGATGCTGAGAACCTAGGC (F)	CATGCTCCACACATTTGAGAAA (R)
*CYP4F3*	AGAAGAAGGGGAGAGGAGGTTGTG (F)	GAGGCGGCAGCAGTTGTCATAG (R)
*CYP4F12*	AAGGCATTGTCAGATGAGGATA (F)	TTCAATCTCTTTAGGATCGCGG (R)
*LPIN1*	GAGCAGCAGAACTCTTCCTAAT (F)	CTTTTGCAATCTACCAGGCTAC (R)
*LDLR*	CTGTAGGGGTCTTTACGTGTTC (F)	GTTTTCCTCGTCAGATTTGTCC (R)
*ACSL1*	CTGTGTCATGGAGCTAAAATCG (F)	GTGTTTGCTTGTCCGAAAATTC (R)
*SCP2*	GGAGCTGAGAATTCAAGAGACT (F)	AGTCACCAAAAACATAGCCAAC (R)
*β-ACTIN*	GCACTCTTCCAGCCTTCCTTCC (F)	GCGGATGTCCACGTCACACTTC (R)

## Data Availability

Data are contained within the article and Supplementary Materials.

## Supplementary Materials

The following supporting information can be downloaded at: https://www.mdpi.com/article/10.3390/toxins16090375/s1, Table S1: The names of the top 20 metabolites with VIP values corresponding to ID numbers.
