# Supplementary material for: Combining Multiple Omics with Molecular Dynamics Reveals SCP2-Mediated Cytotoxicity Effects of Aflatoxin B1 in SW480 Cells
Source: Toxins (Basel). 2024 Aug 24;16(9):375. doi: 10.3390/toxins16090375 (PMC11435460; doi:10.3390/toxins16090375)
Supplement: Supplementary file 1 [file toxins-16-00375-s001.zip › toxins-3113342-supplementary.pdf]

Table S1. The names of the top 20 metabolites with VIP values corresponding to ID numbers.

Index	Compounds
MEDP1055	DL-Glyceraldehyde 3-Phosphate
MEDN0506	N-Acetylglucosamine 1-Phosphate
MW0114958	2-Deoxy-2-Acetamido-Beta-D-Galactose-4-Sulfate
MW0148180	Debromohymenialdisine
MW0123493	Dauricine
MW0159322	Veratridine
MW0106333	Cys-Tyr
MEDN1254	LPI(18:1)
MW0151483	Ile-Leu-Ala-Ile-Val
MW0000410	Psychotrin
MW0170020	Xanthosine
MEDN0428	N6-Succinyl Adenosine
MW0006787	Dichlorprop
MW0127546	Pirinixic acid
MW0061618	[(1S,2R,6S,7R,9R,11R,12S,13S,14S,16R,17S)-16-[(1R)-1-[(2R)-4,5-dimethyl-6-oxo-2,3-dihydropyran-2-yl]ethyl]-6,12-dihydroxy-2,17-dimethyl-3-oxo-8,15-dioxahexacyclo[9.8.0.02,7.07,9.012,17.014,16]nonadec-4-en-13-yl] acetate
MW0004918	2-Dodecylbenzenesulfonic acid
MW0110614	1-Amino-propan-2-ol
MW0125971	(9Z)-N-[2-(5-hydroxy-1H-indol-3-yl)ethyl]octadec-9-enamide
MW0169433	Luteolin
MW0009088	Nilotinib
